# The Rate and Risk Factors of Acute Kidney Injury among Cancer Patients' Admissions in Palestine: A Single-Center Study

**DOI:** 10.1155/2022/2972275

**Published:** 2022-01-12

**Authors:** Zaher Nazzal, Fatima Abdeljaleel, Aseel Ashayer, Husam Salameh, Zakaria Hamdan

**Affiliations:** ^1^Department of Medicine, Faculty of Medicine and Health Sciences, An-Najah National University, Nablus, State of Palestine; ^2^Department of Oncology, An-Najah National University Hospital, Nablus 44839, State of Palestine; ^3^Department of Nephrology, An-Najah National University Hospital, Nablus 44839, State of Palestine

## Abstract

**Introduction:**

Acute kidney injury (AKI) remains a critical issue for cancer patients despite recent treatment improvements. This study aimed to assess the incidence of AKI in cancer patients and its related risk factors.

**Methods:**

A Retrospective cohort study was conducted at tertiary hospitals in the period 2016–2018. A data abstraction sheet was used to collect related variables from patients' records. During admission, the incidence of AKI was assessed using creatinine measurements. RIFLE criteria were used to classify it into five categories of severity: risk, injury, failure, loss, and end-stage renal disease.

**Results:**

Using RIFLE (Risk, Injury, Failure, Loss, and End-stage renal disease) criteria, 6.9% of admissions were complicated with AKI. The severity of these fell into the categories of risk, injury, and failure, 3.3%, 1.7%, and 1.9%, respectively. In the multivariate model, the odds for developing AKI was significantly higher for patients with congestive heart failure (AOR = 17.1, 95% CI 1.7–80.1), chronic kidney disease (adjusted OR = 6.8, 95% CI 1.4–32.2 (*P* value 0.017)), sepsis (AOR = 4.4, 95% CI 1.9–10.1), hypercalcemia (AOR = 8.4, 95% CI 1.3–46.1), and admission to the ICU (AOR = 5.8, 95% CI 2.1–16.2). In addition, the mortality rate was nearly seven times higher for patients complicated by AKI (relative risk = 7.6, 95% CI 3.2–18.2).

**Conclusion:**

AKI was significantly associated with congestive heart failure, chronic kidney disease, sepsis, ICU admission, and hypercalcemia in cancer patients, resulting in poorer outcomes and higher mortality rates. AKI assessment for hospitalized cancer patients should be performed regularly, especially for patients at increased risk.

## 1. Introduction

Treatment of cancer has seen several significant developments in recent years. For many patients, the oncology landscape has dramatically changed and improved [1]. Patients previously thought to have failed treatment are now derived significant advantages with reduced tumor development and improved survival with often less serious systemic medication adverse effects. Despite the apparent positive growth in chemotherapy, drug nephrotoxicity remains a complication and, in some cases, limits life-saving therapy [2].

Cancer patients are at increased risk of acute kidney injury (AKI). Despite the substantial improvement in cancer treatment in recent years, the incidence of AKI among cancer patients remains high. Depending on the type of cancer, two retrospective studies found an average one-year incidence of AKI in cancer patients between 11 and 20% [3, 4]. Another Chinese study recorded 14 to 20 percent AKI incidence depending on the hospital and community versus academic [5].

The high incidence of AKI among cancer patients is multifactorial and attributed to cancer- and patient-specific factors and chemotherapy-induced renal toxicity. Patients with cancer may be at an increased risk of AKI because of malignant infiltration, tumor lysis syndrome, urinary tract obstruction, sepsis, metabolic disturbance, volume depletion, older age (>65 years), female gender, radiation, or nephrotoxic medicinal items. In addition, common cancer comorbidities, such as chronic renal disease, cardiac insufficiency, high blood pressure, diabetes, and liver disease, can also raise the risk [3]. A cohort study of 37,267 Danish cancer patients found that AKI's risk for 1 and 5 years was 17.5% and 27.0%, respectively, with the highest 1-year risk in patients with multiple myeloma (31.8%), liver cancer (33.0%), and kidney cancer (44.0%), respectively [3]. Others reported that 12% of hospitalized patients developed AKI using RIFLE criteria. Also, 12 to 49% of critically ill cancer patients developed AKI during the intensive care unit (ICU) stay, and 9 to 32% of them needed kidney replacement therapy [6, 7].

AKI is associated with significant morbidity and mortality in cancer patients, including high mortality, increased hospitalization, and a lower cancer remission [8–12]. Short- and long-term outcomes of AKI in cancer patients are poor, with just 14% in one study with a 60-day survival rate [8]. Other studies show that AKI has a 3-month mortality rate of over 30% [13] and a 6-month mortality rate of up to 73% in cancer patients [14]. Also, 5.1% of patients with AKI had long-term dialysis within one year [8].

AKI can adversely affect cancer treatment outcomes by minimizing the dosage of chemotherapy, reducing the likelihood of full recovery, increasing hospital frequency and duration, and decreasing functionality and quality of life without any additional cancer therapy [15]. For example, within one year of AKI initiation, 5% of patients in the large Danish study needed renal replacement therapy [3]. This rate increased to 60% among critically ill, higher-risk patients, depending on AKI's severity and underlying comorbidities [6].

AKI prevention is the most crucial and essential AKI control strategy for cancer patients [16]. It relies on assessing the burden of the problem and recognizing patient- and cancer-specific risk factors that may be targeted for intervention to reduce the likelihood of AKI. Therefore, this study's objective was to assess AKI incidence in cancer patients in a tertiary teaching hospital and establish their link with the patient- and cancer-specific risk factors.

## 2. Materials and Methods

### 2.1. Study Design and Population

A retrospective chart review study was conducted at the AL-Najah National University Hospital (NNUH), a JCI-approved tertiary healthcare facility that receives almost all cancer patients from the West Bank and Gaza. All patient records from January 2016 to January 2018 were included in the analysis. The study population included patients over the age of 16 with solid tumors or multiple myeloma. Eligibility for inclusion in this analysis was any patient admitted to the medical unit during the study period with serum creatinine at admission and further readings during a hospital stay. Hospital admission was defined as a hospital stay of ≥24 hours. Bone marrow transplant patients and outpatient patients were excluded.

Each patient's demographics, medical conditions, laboratory findings, medications, treatment outcomes, and ICU admissions have all been collected. Medical conditions included antibiotic use, chemotherapy, blood pressure, congestive heart failure, chronic renal illness, multiple malignancies, sepsis, ICU admissions, nephrectomy, hypercalcemia, and death.

The Institutional Review Board (IRB) of Al-Najah National University ethically approved the study. Also, permission to conduct it at the oncology department was obtained from NNUH. The information gathered was used solely for research purposes and was kept strictly confidential.

### 2.2. AKI Definition and Staging

A data abstraction sheet was developed and used for the collection of relevant variables. It collects information from patients' records filled out by hospital residents, physicians, and pathology and radiology departments. Incident of AKI after admission was assessed using creatinine measurements recorded in laboratory databases. These databases included the time and result of all analyses performed in the hospital laboratory. The baseline creatinine level was the creatinine level at the time of admission, and the second reading was the highest reading during the same admission.

We used RIFLE criteria to classify AKI into five categories of severity: risk, injury, failure, loss, and end-stage renal disease [17]. Cancer patients with a 50% increase in creatinine compared to their baseline levels were considered AKI in one of these five categories [17]. The category injury corresponds to a 2-fold increased creatinine level and failure to a 3-fold high creatinine or a ≥350 *μ*mol/l with an acute rise ≥of 44 *μ*mol/l.

### 2.3. Statistical Analysis

Version 20 of SPSS software was used for data entry and analysis. Tables and figures presented patient characteristics, and the rate of AKI was calculated. The AKI rates were compared between different groups by the *t*-test or chi-squared tests. *P* values <0.05 (two-tailed) were regarded as statistically significant. A logistic regression model was used to analyze the association between all-cause in-hospital AKI and relevant covariates. Odds ratios with 95% confidence intervals and *P* values of the Wald chi-square test were reported.

## 3. Results

Eight hundred and twenty admissions (204 patients) were reviewed in 2 years (2016 and 2018). Eighty-two admissions (27 patients) were excluded due to missed creatinine readings. Of the 200 patients (638 admissions), 48.4% were males, and the mean age was 54.7 ± 12.9 years (Table 1). Among the studied sample, 63 (31.5%) had a history of diabetes, 59 (29.5%) had hypertension, 23 (11.5%) had ischemic heart disease, and 7 (3.5%) had chronic kidney disease. Also, 112 patients (56%) had distant metastases, 44 had liver cancer (22%), and 11(5.5%) had multiple cancers.

Based on RIFLE criteria, 44(6.9%) admissions during their hospital stay were complicated by AKI. The risk, injury, and failure severity categories were 22 (3.3%), 11 (1.7%), and 12 (1.9%), respectively. There was an AKI episode every 11.8 days spent in the hospital.

Univariate analysis was performed to investigate the factors linked to the development of AKI episodes. The frequency of AKI was found to be significantly higher in patients with HTN, CKD, CHF, and multiple cancers and those who had a history of nephrectomy due to renal cell cancer (*P* value <0.05) (Table 1).

For the clinical characteristics, the frequency of AKI was significantly higher in patients who received antibiotics, received chemotherapy, had hypercalcemia, developed sepsis, and were admitted to the ICU (*P* value <0.05) (Table 2).

There have been recorded deaths in the hospital for 33 patients. The in-hospital mortality rate for the entire study admissions was 16.2% (33/204 patients). Fourteen (48.3%) of the AKI-complicated patients died. Patients complicated by AKI are nearly seven times more likely to result in death than non-AKI patients (*P* value < 0.001, relative risk = 7.6, 95% CI 3.2–18.2). Moreover, the in-hospital length of stay and ICU admissions were significantly higher among the admissions complicated by AKI (*P* value <.001) (Table 3).

We carried out a multivariable logistic regression analysis to control confounders and assess AKI risk factors. The model included all significant variables in the univariable analysis (*P* value <0.05). The findings demonstrated that the occurrence of AKI was strongly associated with congestive heart failure (adjusted OR = 17.1, 95% CI 1.7–80.1 (*P* value 0.015)), chronic kidney disease (adjusted OR = 6.8, 95% CI 1.4–32.2 (*P* value 0.017)), sepsis (adjusted OR = 4.4, 95% CI 1.9–10.1 (*P* value <0.001)), hypercalcemia (adjusted OR = 8.4, 95% CI 1.3–46.1 (*P* value 0.015)), and admission to the ICU (adjusted OR = 5.8, 95% CI 2.1–16.2 (*P* value 0.001)) (Table 4).

## 4. Discussion

Cancer and its treatment can be associated with multiple AKI-inducing events [18]. The overall incidence of AKI in cancer patients is estimated to be higher than the rate recorded for noncancer settings [19–21].

Our study revealed that 6.9% of cancer patients' admissions had AKI based on the adjusted RIFLE criteria. The distribution of patients with any degree of AKI was risk (3.3%), injury (1.7%), and failure (1.9%). Previous research has shown that AKI was seen in 12% to 66.5% of cancer patients [4, 14, 22–24], higher than AKI in our sample. Many of these studies are limited to selected high-risk AKI groups, such as hematological malignancies [22] and surgical-treated patients [24] and patients in the ICU [23]. In our study, we included all cancer patients admitted regardless of their primary diagnosis at the time of admission. Also, the frequency of AKI depends on the definition of creatinine's baseline level, which causes interstudy heterogeneity [25]. Many retrospective studies use low or admission serum creatinine as the baseline value [4, 22, 23], which may be sensitive to AKI detection and overestimate the incidence. In this study, only patients with at least two creatinine readings were considered during hospitalization, and a complex definition of baseline value was used; this method makes estimates more reliable.

The etiology of AKI in hospitalized cancer patients is often multifactorial [14, 26], and our study has identified several independent AKI factors. In the adjusted model, AKI incidence was significantly related to congestive heart failure, chronic kidney disease, sepsis, ICU admission, and hypercalcemia. In addition, according to outcome analyses, AKI in cancer patients was associated with higher odds for deaths and more extended hospital stays.

AKI is 4.4 times more likely to develop among sepsis cancer hospitalizations compared to sepsis-free hospitalizations. The kidney is one of the most frequently affected organs after sepsis, causing AKI-related sepsis and contributing to sepsis morbidity and mortality [27]. This can be due to renal hypoperfusion, DIC, multisystem failure, and increased ICU admission probability [28].

In this study, the development of AKI was found to be 5.5, more likely among cancer hospitalizations admitted to the ICU. Admission to the ICU was found to be a contributing factor to the development of AKI [29]. This may be due to acute tubular necrosis, deeper hypotension, multiorgan failure, and increased hospital stay.

Some comorbidities have been shown to be strongly associated with AKI, chronic kidney disease, and congestive heart failure. Chronic kidney disease is characterized by chronic inflammation and vascular dysfunction, and uncontrolled congestive heart failure is associated with a rapid loss of renal function. These pathological changes are contributing to an increase in the AKI rate. Moreover, congestive heart failure and chronic kidney disease tend to work together as a vicious cycle in which each condition causes and exacerbates the other.

Hypercalcemia occurs in approximately 20% to 30% of all malignancies and is a common cause of AKI [21]. In this study, hypercalcemia was a strong and independent clinical association with AKI. In addition, there is an important correlation between hypercalcemia and poor health outcomes in cancer patients, suggesting hypercalcemia as a potential marker for sicker patients.

Our patients' mean age was 55 years, and age in the multivariate analysis did not differ between the AKI and non-AKI groups. This finding suggests that factors other than age were important for AKI in our patients with cancer. In contrast to our analysis, many studies reported that chemotherapy is a significant factor in developing AKI [4, 30]. This was not found in our research, and the negative correlation may be secondary to preventive measures taken when chemotherapy was administered.

The occurrence of AKI was found to predict mortality in hospitalized cancer patients [20, 29, 31, 32]. Our analysis found that the in-hospital mortality rate was 5.2% for all cancer hospitalizations. Of these, 39.3% (13 patients) were complicated with AKI. Furthermore, a study conducted by Liborio et al. showed that mortality was significantly higher in AKI cancer patients than in non-AKI patients and found that the risk of mortality increased along with the increase in AKI severity category [29].

The key strength of the study is its population-based hospital setting within a free health insurance-supported healthcare system. While our study included large sample size and collected detailed information on risk profiles, some limitations warrant consideration. First, urine output was not recorded for all patients, and thus, only the change in serum creatinine was used in the RIFLE criteria of AKI. As a result, some patients with AKI with only a transient reduction in urine output and no serum creatinine change were not accounted for. However, our estimates are likely to be conservative, as a 50% rise in creatinine, the lowest category we assessed, is correlated with a markedly reduced glomerular filtration rate. The second is the lack of follow-up data, as we could not examine long-term mortality and CKD development. Third, the poor data reporting did not enable us to assess variables such as tobacco use, albumin, urine output, need for pressors, and need for ventilator as they were missed for many patients. Finally, the case mix of malignancies, the cause of hospitalization, and using specific but nonsensitive criteria may have impacted the reported incidence of AKI.

In conclusion, the rate of AKI in hospitalized cancer patients is high. Regardless of the underlying cancer, the risk of developing AKI during hospitalization was higher for congestive heart failure patients, chronic kidney disease, hypercalcemia, sepsis, and ICU admission. In addition, AKI was associated with a higher mortality rate in hospitalized patients. Therefore, studies are justified in assessing whether preventive AKI measurement can reduce AKI occurrence, especially in high-risk cancer patients, and enhance clinical outcomes.

## Figures and Tables

**Table 1:** Univariable analysis of AKI with cancer patients' background characteristics and comorbidities.

Variables	Total frequency (%)	AKI +ve frequency (%)	AKI −ve frequency (%)	*P* value^*∗*^
Gender				
Male	309 (48.4%)	21 (6.8%)	288 (93.2%)	0.923
Female	329 (51.6%)	23 (7%)	306 (93%)	
Age in years (mean ± SD)	54.7 ± 12.9	58 ± 7.5	54.5 ± 13.2	0.082^*∗∗*^
Diabetes				
Yes	135 (21.2%)	8 (5.9%)	127 (94.1%)	0.616
No	503 (78.8%)	36 (7.2%)	467 (92.8%)	
Hypertension				
Yes	97 (15.2%)	15 (15.5%)	82 (84.5%)	<0.001
No	541 (84.4%)	29 (5.4%)	512 (94.6%)	
Ischemic heart disease				
Yes	34 (5.3%)	2 (5.9%)	32 (94.1%)	0.810
No	604 (90.7%)	42 (7%)	562 (93%)	
Obstructive lung disease				
Yes	11 (1.7%)	2 (18.2%)	9 (81.8%)	0.136
No	627 (98.3%)	42 (6.7%)	585 (93.3%)	
Benign prostatic hyperplasia				
Yes	9 (1.4%)	0	9 (100%)	0.411
No	629 (98.6%)	44 (7%)	585 (93%)	
Chronic kidney disease				
Yes	14 (2.2%)	6 (42.9%)	8 (57.1%)	<.001
No	624 (97.8%)	38 (6.1%)	586 (93.9%)	
Hepatitis B				
Yes	9 (1.4%)	2 (22.2%)	7 (77.8%)	.68
No	629 (98.6%)	42 (6.7%)	587 (93.3%)	
Distant metastasis				
Yes	414 (64.9%)	24 (5.8%)	390 (94.2%)	.136
No	224 (35.1%)	20 (8.9%)	204 (91.1%)	
History of nephrectomy				
Yes	17 (2.7%)	4 (23.5%)	13 (76.5%)	.006
No	621 (97.3%)	40 (6.4%)	581 (93.6%)	
Congestive heart failure				
Yes	4 (0.6%)	2 (50%)	2 (50%)	.001
No	634 (99.4%)	42 (6.6%)	592 (93.4%)	
Multiple cancers				
Yes	12 (1.9%)	4 (33.3%)	8 (66.7%)	<.001
No	626 (98.1%)	40 (6.4%)	586 (93.6%)	
Retroperitoneal disease				
Yes	29 (4.5%)	1 (3.4%)	28 (96.6%)	.453
No	609 (95.5%)	43 (7.1%)	566 (92.9%)	

^*∗*^Chi-square test. ^*∗∗*^Independent *t*-test.

**Table 2:** Univariate analysis of AKI with clinical characteristics among cancer patients during admissions.

Variables	Total frequency (%)	AKI +ve frequency (%)	AKI −ve frequency (%)	*P* value^*∗*^
IV contrast use				
Yes	80 (12.5%)	4 (5%)	76 (95%)	.474
No	558 (87.5%)	40 (7.2%)	518 (92.8%)	
Antibiotic use				
Yes	250 (39.2%)	31 (12.4%)	219 (87.6%)	<.001
No	388 (60.8%)	13 (3.4%)	375 (96.6%)	
Chemotherapy use				
Yes	320 (50.2%)	13 (4.1%)	301 (95.9%)	.015
No	315 (49.5%)	31 (9.8%)	284 (90.2%)	
Radiotherapy				
Yes	24 (3.8%)	2 (8.3%)	22 (91.7%)	.777
No	614 (96.2%)	42 (6.8%)	572 (93.2%)	
Hypercalcemia				
Yes	7 (1.1%)	3 (42.9%)	4 (57.1%)	<.001
No	631 (98.9%)	41 (6.5%)	590 (93.5%)	
Sepsis				
Yes	90 (14.1%)	20 (22.2%)	70 (77.8%)	<.001
No	548 (85.9%)	24 (4.4%)	524 (95.6%)	

*∗*Chi-square test.

**Table 3:** Acute kidney injury outcomes.

Variables	Total	AKI +ve	AKI −ve	*P* value^*∗*^
In-hospital mortality†				
Yes	33 (16.2%)	14 (48.3%)	19 (10.9%)	
No	171 (83.8%)	15 (51.7%)	156 (89.1%)	<.001^*∗*^
ICU admission				
Yes	28 (4.4%)	10 (35.7%)	18 (64.3%)	
No	610 (95.6%)	34 (5.6%)	578 (94.4%)	<.001^*∗*^
Length of stay (mean ± SD)		13.57 ± 13.1	5.28 ± 6.6	<.001^*∗∗*^

†Mortality is calculated per patient. ^*∗*^Chi-square test. ^*∗∗*^Independent *t*-test.

**Table 4:** Logistic regression analysis for factors associated with AKI in patients admitted.

Risk factors	Multivariable analysis		
	Adjusted OR	(95% CI)	*P* value
Age	0.99	(.96–1.1)	0.754
Antibiotics	1.59	(0.76–4.9)	0.166
Chemotherapy	0.77	(0.32–1.6)	0.714
Hypertension	1.6	(0.65–3.8)	0.311
Congestive heart failure	17.1	(1.7–80.1)	0.015
Chronic kidney disease	6.8	(1.4–32.2)	0.017
Multiple cancers	4.1	(0.63–26.1)	0.153
Sepsis	4.4	(1.9–10.1)	<0.001
ICU admission	5.8	(2.–16.2)	0.001
History of nephrectomy	3.2	(0.58–17.6)	0.319
Hypercalcemia	8.4	(1.3–46.1)	0.028

## Data Availability

The data used to support the findings of this study are available from the corresponding author upon request.
